# Screening Utility, Local Perceptions, and Care-seeking for Reported *Jaundeesh* among Respondents Lacking Signs of Icterus in Rural Bangladesh

**DOI:** 10.3329/jhpn.v31i3.16829

**Published:** 2013-09

**Authors:** Mohammad Z. Hossain, Shegufta S. Sikder, K. Zaman, Parimalendu Saha, Mohammad Yunus, Kenrad E. Nelson, Alain B. Labrique

**Affiliations:** ^1^icddr,b, GPO Box 128, Dhaka 1000, Bangladesh; ^2^Department of International Health, Johns Hopkins Bloomberg School of Public Health, 615 N. Wolfe St., Baltimore, MD 21205, USA; ^3^Department of Epidemiology, Johns Hopkins Bloomberg School of Public Health, 615 N. Wolfe St., Baltimore, MD 21205, USA

**Keywords:** Ethnography, Hepatitis, HEV, Jaundice, Medical anthropology, Morbidity, Traditional healers, Bangladesh

## Abstract

In rural Bangladesh, acute viral hepatitis presents a significant burden on the public-health system. As part of the formative work for a large epidemiologic study of hepatitis E in rural Bangladesh, we sought to identify local terms that could be used for population-based screening of acute viral hepatitis. Exploration of the local term *jaundeesh* for screening utility identified a high burden of reported *jaundeesh* among individuals without symptoms of icterus. Recognizing that local perceptions of illness may differ from biomedical definitions of disease, we also sought to characterize the perceived aetiology, care-seeking patterns, diagnostic symptoms, and treatments for reported *jaundeesh* in the absence of icteric symptoms to inform future population-based studies on reported morbidities. We conducted a cross-sectional survey among 1,441 randomly-selected subjects to identify the prevalence of reported *jaundeesh* and to test the validity of this local term to detect signs of icterus. To characterize the perceived aetiology and care-seeking patterns for *jaundeesh* among the majority of respondents, we conducted in-depth interviews with 100 respondents who self-reported *jaundeesh* but lacked clinical signs of icterus. To describe diagnostic symptoms and treatments, in-depth interviews were also performed with 25 *kabirajs* or traditional faith healers commonly visited for *jaundeesh.* Of the 1,441 randomly-selected participants, one-fourth (n=361) reported *jaundeesh*, with only a third (n=122) reporting yellow eyes or skin, representative of icterus; *Jaundeesh* had a positive predictive value of 34% for detection of yellow eyes or skin. Anicteric patients with reported *jaundeesh* perceived their illnesses to result from humoral imbalances, most commonly treated by amulets, ritual handwashing, and bathing with herbal medicines. *Jaundeesh* patients primarily sought folk and spiritual remedies from informal care providers, with only 19% visiting allopathic care providers. Although the local term *jaundeesh* appeared to have limited epidemiologic utility to screen for acute symptomatic viral hepatitis, this term described a syndrome perceived to occur frequently in this population. Future population-based studies conducting surveillance for acute hepatitis should use caution in the use and interpretation of self-reported *jaundeesh.* Further study of *jaundeesh* may provide insight into the appropriate public-health response to this syndrome.

## INTRODUCTION

Viral hepatitis is a significant public-health problem in Bangladesh (1). Although hepatitis A and E viruses (both water-borne, faecal-oral pathogens) cause low mortality in the general population, these lead to significant annual morbidity and loss of productivity across much of South Asia (1,2). Exposure to hepatitis A is nearly ubiquitous, resulting in widespread infection in early life and consequent lifelong immunity (1). Assessing clinical data, 1,823 patients in Dhaka, Bangladesh, suspected to suffer from hepatitis B, Khan *et al.* detected antibodies to hepatitis A and E virus among 39% and 53% of subjects respectively. Hepatitis E, associated with elevated case-fatality ratios in pregnant women (up to 20%) (3), is considered a major public-health problem across most of South Asia and has been shown to contribute up to 10% of pregnancy-related deaths in rural northwest Bangladesh (4,5). Despite this burden, this emerging pathogen remains significantly under-studied and under-recognized as an aetiologic agent in this population (3,6). The ubiquitous risk of exposure to faecal-oral pathogens has been well-documented across rural and urban populations of the country, explaining the elevated burden of viral hepatitis infections (3,6).

Improved contextual understanding of specific diseases in endemic populations is essential to optimize surveillance, control, and targeted care. In settings where clinical consultations may be challenging, population-based surveys seek to elicit reported symptoms and morbidities during routine surveillance. For detection of acute viral hepatitis, population-based surveys often assess report of clinically-proven jaundice, also known as icterus (the yellowing of the eyes or skin as a result of hyperbilirubinaemia or liver cholestasis—a sign of liver distress or damage) as a symptom that triggers further clinical investigation for presence of hepatitis (7). While local terms may be useful in detecting symptoms warranting further investigation, studies in rural Bangladesh have suggested that local perceptions of illness may differ from biomedical definitions (8-11). For conditions ranging from infectious and chronic diseases to pregnancy-related illnesses, studies have described cultural frameworks of perceived illness aetiology, which influence conceptions of disease as well as subsequent care-seeking behaviours (8-12). Despite the documented elevated burden of viral hepatitis, little has been reported about the medical anthropology of this spectrum of infections, including local perceptions and practices, care-seeking behaviour, and treatments.

In Bangladesh, perceived aetiologies of illness have been linked to care-seeking behaviours (8-10,13). Studies from Bangladesh reveal a plural health system that consists of both informal (defined as care providers lacking formal certification) and formal care providers (defined as healthcare providers who are recognized and regulated by legal authorities), such as doctors, nurses, midwives, or government-trained care providers (8-10,13). For illnesses, including some that are typically believed to arise from non-medical causes, rural residents have reported visiting informal care providers, such as traditional healers, village doctors, shamans, traditional birth attendants, and homeopathic doctors (14). Traditional healers, including shamans, are generally visited for conditions that are perceived to be caused by evil spirits, and they provide blessings against these spirits (15). In Bangladesh, a common traditional healer is called *kabiraj*, a healthcare provider who practises a combination of ayurvedic medicine and faith healing (15). Allopathic care providers include village doctors and homeopathic care providers, some with training or apprenticeship experience, who provide allopathic treatments for various ailments in rural communities (9,16). Although studies reveal the importance of informal care providers in care-giving for numerous illnesses, care-seeking for hepatitis and hepatitis-like illnesses has not been well-described in this rural population. A recent large outbreak of hepatitis E in an urban population of Bangladesh nearly went undetected by the public health sector (Gurley. Personal Communication, 2010) as most of the cases did not seek formal care for their condition. Although understanding care-seeking pathways is critical to developing effective public-health strategies for prevention and treatment (10), data on perceptions of jaundice in rural communities are lacking.

As part of the formative research for a large epidemiologic study of hepatitis E in rural Bangladesh, we sought to test the validity and utility of local terms tested and validated elsewhere in the country, to detect clinical signs of icterus in this population. The frequent report of the local term *jaundeesh* was initially a promising finding, given the strong pathognomonic association of scleral and dermal icterus with acute hepatitis (7). However, formative research suggested a high burden of reported *jaundeesh* among respondents without symptoms of icterus (6). Recognizing that local illness perceptions may differ from biomedical definitions of disease, we also sought to characterize the perceived aetiology, care-seeking patterns, diagnostic symptoms, and treatments for reported *jaundeesh* in the absence of icteric symptoms to inform future population-based studies on reported morbidities.

## MATERIALS AND METHODS

The study was conducted in Matlab (a subdistrict approximately 55 km southwest of Dhaka) where the International Centre for Diarrhoeal Disease Research, Bangladesh (icddr,b) has been implementing a health and demographic surveillance system in a population of about 220,000 for the past four decades (17). Since 1966, community health research workers (CHRWs) have collected vital statistics, such as on marriage, birth, death, and migration among this population.

This three-phase study was conducted as part of the formative research preceding a large longitudinal study which sought to identify age-specific incidence of hepatitis E infections under endemic, non-outbreak conditions in rural Bangladesh (3). The formative research, completed in 2003, was conducted in three phases to achieve the three research objectives. From the 2003 Census of the Matlab Health and Demographic Surveillance System (HDSS), a random list of 1,450 individuals was generated for inclusion. Children aged less than one year were not sampled due to the reluctance of parents to allow blood-draws from healthy young infants.

From the selected 1,450 individuals, 1,441 consented for hepatitis surveillance and were included in this study. Thirteen CHRWs regularly visited households to ask participants if, in the last 30 days, they or anyone in their family experienced any of the following six hepatitis-like symptoms: (i) nausea or vomiting, (ii) fever and severe weakness, (iii) loss of appetite or foul smell in food, (iv) yellow eyes or skin, (v) ash-coloured stool or tea-coloured urine, or (vi) *jaundeesh*/*holde palong*. Pre-testing of these questions revealed that the local terms *jaundeesh* or *holde palong* were sometimes used for describing conditions consistent with biomedical jaundice, these terms were used in the screening algorithm at the outset of the study. If respondents reported experiencing any symptoms, the duration of each reported symptom was recorded.

If participants reported experiencing *jaundeesh*/*holde palong* lasting between eight and 14 days, the CHRWs sent their information to a medical team (consisting of an MBBS doctor and a laboratory technician) to schedule an examination. A blood sample was collected if physicians found the participant to exhibit visible signs consistent with clinical icterus. For practical purposes, only those respondents who had visible icterus, defined as yellow eyes or skin confirmed by a medical officer, were approached for blood samples. The medical teams used the symptoms of yellow eyes or skin for icterus due to greater practicality of confirming these symptoms during home-visits compared to symptoms, such as ash-coloured stool or tea-coloured urine. In addition, the symptoms of dark-coloured urine or light clay-coloured stools have been considered to be less reliable for detection of hepatitis compared to the symptoms of yellow eyes or skin (18).

The positive predictive value and negative predictive value of the terms *jaundeesh/holde palong* to identify patients with yellow eyes or skin were calculated. Data from this process led to further refinement of the screening algorithm and the eventual exclusion of *jaundeesh/holde palong.* Findings from the larger study, published elsewhere, showed that most of the tested participants did not exhibit acute-phase antibodies to hepatitis A, B, C, or E (3).

Recognizing the large burden of patients who reported *jaundeesh/holde palong* in this population in the absence of visible signs of icterus, we sought to further characterize these local terms by describing their common perceived aetiology, diagnostic symptoms, and treatments to inform future population-based studies. The second phase of formative research consisted of in-depth interviews with100 participants, randomly selected from respondents who self-reported *jaundeesh* and who lacked symptoms of yellow eyes or skin. Two field research assistants, both with five years of experience implementing field interviews in Matlab and with training on qualitative methods, conducted in-depth interviews, using a structured questionnaire. The field research assistants asked respondents to describe their understanding of how their *jaundeesh* was acquired, the symptoms that they associated with this condition, and their typical treatment-seeking behaviour.

Respondents were asked about the healthcare providers from whom they typically sought treatment for themselves or their family members. Since *kabirajs* were the most commonly-visited treatment providers, the interview team asked respondents about the name and location of the *kabirajs* that they visited. Using this list for the third stage of formative research, the team conducted in-depth interviews with the 25 most commonly-visited traditional healers or *kabirajs* in the Matlab study area to understand how these care providers diagnosed *jaundeesh* and the treatments they commonly prescribed. The interview team asked the *kabirajs* about their age, years of experience, weekly number of patients seen, and diagnostic symptoms and treatments provided for *jaundeesh/holde palong.*

All quantitative data were analyzed in Excel 2010 to calculate sensitivity, specificity, positive predictive value, and negative predictive value of the term *jaundeesh* to detect symptoms of yellow eyes or skin. For the qualitative data from in-depth interviews, a medical officer and a senior field research officer assisted the field research assistants with transcription. Using Microsoft Word, the in-depth interviews were reviewed and coded line-by-line to identify common themes described as constituting *jaundeesh*. These data were used in producing a conceptual framework of aetiology of perceived jaundice in the study population. Quantitative data on care providers sought and their typical treatments were analyzed in Excel 2010 to produce frequency distributions of care providers most frequently approached, diagnostic symptoms, and common treatments. Study procedures were approved by the Committee on Human Research at the Johns Hopkins Bloomberg School of Public Health and by Ethical Review Committee of icddr,b.

## RESULTS

### Prevalence and screening utility of reported ***jaundeesh***

Of the 1,441 people randomly selected for hepatitis surveillance, 361 (25%) reported having *jaundeesh* while 1,080 people (75%) reported that they did not have *jaundeesh*. Among the 361 people reporting *jaundeesh*, 84% reported loss of appetite or foul smell in food while 68% reported fever or severe weakness, and 51% reported nausea or vomiting (Table 1). Only one-third of the respondents with *jaundeesh* (34%) reported having yellow eyes or skin while 27% reported ash-coloured stool or tea-coloured urine.

**Table 1.** Hepatitis-like symptoms reported among 361 respondents who self-reported *jaundeesh* in Matlab, Bangladesh, in 2003

Symptom	Number (percentage) reporting symptoms among respondents who self-reported *jaundeesh/holde palong* (n=361)*
Loss of appetite and foul smell in food	304 (84)
Fever and severe weakness	245 (68)
Nausea or vomiting	185 (51)
Yellow eyes or skin	122 (34)
Ash-coloured stool or tea-coloured urine	99 (27)

*The total is greater than 100% since patients could list multiple symptoms

Table 2 shows the positive predictive value of the term *jaundeesh*/*holde palong* to detect yellow eyes or skin. The positive predictive value of the terms *jaundeesh*/*holde palong* for detection of yellow eyes or skin was only 34%, with only 122 out of 361 respondents with *jaundeesh* reporting yellow eyes or skin (Table 2). The negative predictive value of the terms was relatively high since among those not reporting *jaundeesh/holde palong*, 99% (n=1,071) did not report yellow eyes or skin. Although 93% of respondents with yellow eyes or skin reported *jaundeesh* (Table 2), the term *jaundeesh* had limited screening utility to effectively capture icterus, primarily due to the dilution of this pool with a large number of individuals without visible symptoms of yellow eyes or skin.

**Table 2.** Validity values of self-reported *jaundeesh* for detection of visible signs of icterus (yellow eyes or skin) among 1,441 respondents in Matlab, Bangladesh, in 2003

		Yellow eyes or skin		
		Yes	No	Total
*Jaundeesh/holde palong*	Yes	122	239	361
	No	9	1,071	1,080
	Total	131	1,310	1,441
Sensitivity: 93%				
Specificity: 82%				
Positive predictive value: 34%				
Negative predictive value: 99%				

### Perceptions of ***jaundeesh*** among those lacking visible symptoms of icterus

**Figure 1.**
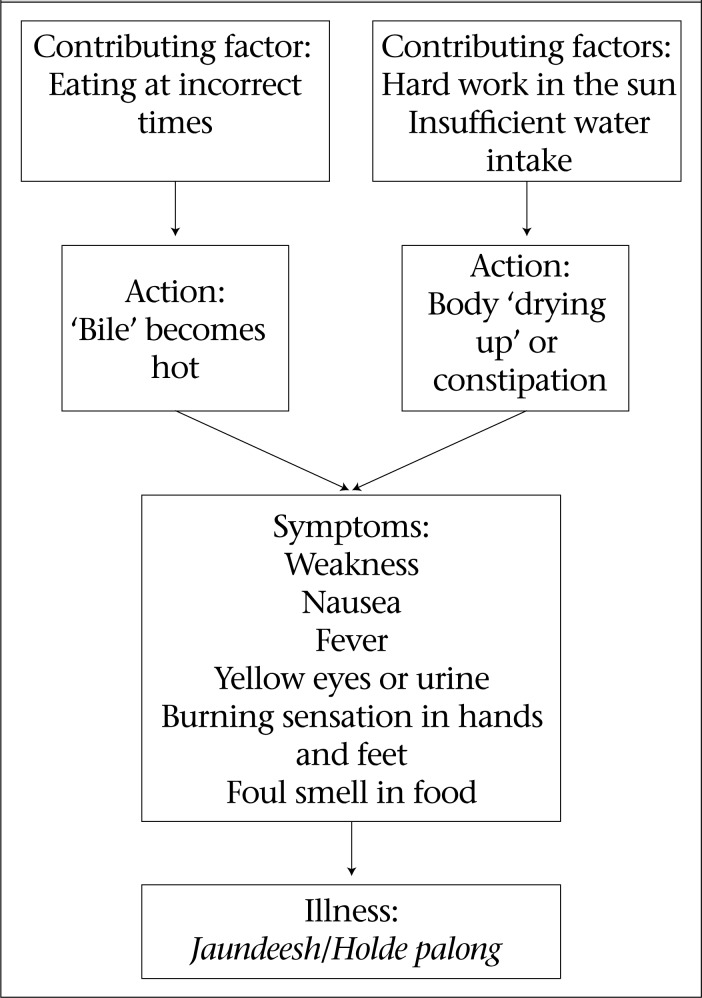
Explanatory framework of pathways to *jaundeesh* from in-depth interviews with 100 anicteric participants reporting *jaundeesh* and lacking clinical icterus in Matlab, Bangladesh, in 2003

Field research assistants conducted in-depth interviews with 100 randomly-selected respondents to understand local perceptions of *jaundeesh* among anicteric respondents. Figure 1 presents a framework of how *jaundeesh* is commonly believed to be manifested, based on common responses. Respondents mentioned eating at incorrect times, insufficient water intake, and hard work in the sun contributing to humoral imbalances, such as bile (local term: *pitta*) becoming hot or the body ‘drying up’ and constipation. Symptoms described as indicative of *holde palong* included a burning sensation in hands and feet, fever, foul smell in food or water, anorexia, weakness, yellow eyes or urine, and nausea. Ranu, a 33-year old woman, explained the process of jaundice acquisition:

Village people say when the body dries up, when the hands and feet burn, stool hardens, and urination decreases, *jaundeesh* happens. Then, many people bathe in their ponds two to three times a day for at least an hour. When the body gets cold, your nose starts to run, then the *jaundeesh* gets better.

Mahfuza, a 40-year old woman, explained a similar process:

Because *jaundeesh* happens from constipation, it means the body, hands, and feet burn, stool becomes hard and ashy. This is why, in the village, those who have *jaundeesh* bathe every hour in the pond. When it is a child, they bathe him [or her] three to four times. They [people with *jaundeesh*] walk around without shoes, so that they get cold. When you get cold, your jaundice is cured.

Most people believed that they had *jaundeesh* due to symptoms, such as loss of appetite, fever, or nausea. Rima, a 30-year old mother who reported *Jaundeesh* in her 3-year old son, explained:

Last monsoon, my son had *jaundeesh.* When I fed him, he would act nauseated and sometimes would vomit. He had fever, and his stool was hard. He didn't want to eat anything. When I saw these symptoms, I realized my son had *jaundeesh*.

Aklima, a 29-year old female, describes similar symptoms:

When I was sick, I had fever and nausea. I could not eat anything; everything had a smell of fish. I had a burning sensation in my hands and feet. Then, I realized I had *jaundeesh*.

Care-seeking for *jaundeesh* among those lacking visible symptoms of icterus

**Figure 2.**
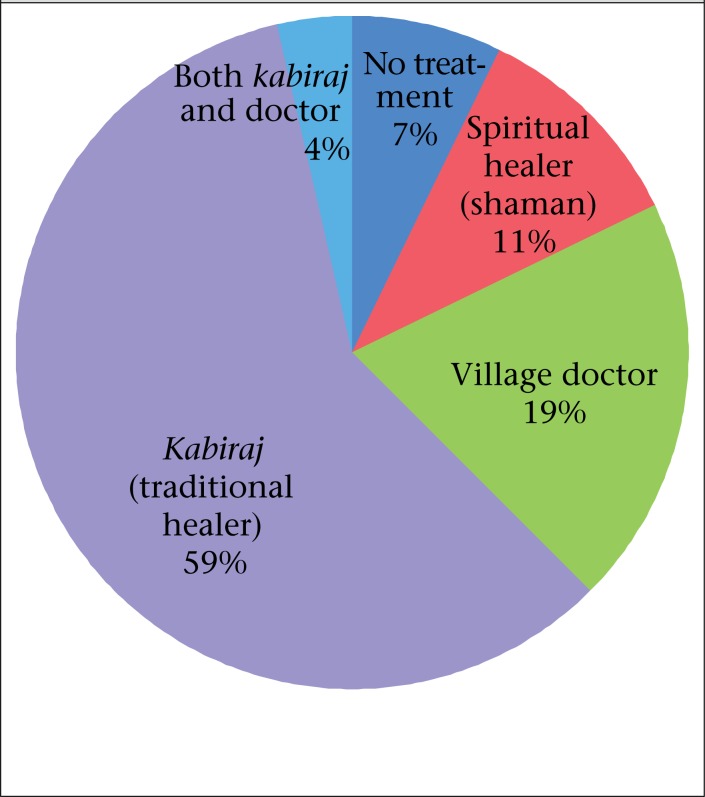
Treatment sought by 100 respondents reporting *jaundeesh* and lacking clinical icterus in Matlab, Bangladesh, in 2003

Figure 2 shows that 59% of the respondents sought care from traditional treatment providers called *kabiraj* while 19% went to village doctors, and 11% visited shamans, or spiritual healers. Seven percent sought no treatment while the remaining 4% sought treatment from both a *kabiraj* and an MBBS (board-certified) doctor.

From the in-depth interviews, respondents described visiting *kabirajs* since they believed the treatments would cure their *jaundeesh*.

Shanti, a 26-year old woman with *jaundeesh* said:

I believe that treatment from *kabiraj* is the best for *jaundeesh.* When I had *jaundeesh*, the *kabiraj* told me to eat blessed bananas for four days. After clean-shaving the top of my head, the *kabiraj* applied herbal paste on my scalp, which I kept for seven days. I also took herbal tablets three times a day for one month, and my *jaundeesh* was cured.

Respondents who sought care from both *kabirajs* and MBBS doctors said they did so due to the lack of specific treatments provided by allopathic doctors. Many of the respondents were pregnant and were not given specific treatments from their doctors.

Jharna, a 23-year old woman, explained:

When I realized I had *jaundeesh*, my father-in-law and husband took me to the hospital. As I was at the seventh month of my pregnancy, they did not give me any medicine and told me to go home and rest. When I came home, everyone told me to see the *kabiraj* for treatment. The *kabiraj* clean-shaved the top of my head and applied herbal paste. For three days, I went to his house so he could wash my hands in ‘root-water’ and place an amulet on my neck. After one and a half months, my symptoms were gone.

Parveen, 27-year old woman, described:

When I had *jaundeesh*, I went to the hospital. They gave me no treatment since I was pregnant and told me to drink lots of fluids. I went to the *kabiraj* who rubbed my hands with branches and leaves and then washed them. With his treatment, my *jaundeesh* disappeared in three months. Some *kabirajs* do handwashing, others give blessed bananas, and some others give amulets to wear. This is the way *jaundeesh* is cured.

Others went to *kabirajs* because they received greater satisfaction from their treatment compared to allopathic treatment. Mother of Abu Bakr, a 3-year old boy with reported *jaundeesh* said:

In the hospital, the doctors did blood tests and said my son had *jaundeesh*. They gave him vitamin drops and told us to come back in four days. I did not go back since my child was not improving with hospital treatment. Instead, I took my child to a *kabiraj*. The *kabiraj* fed my son one blessed banana every morning for three days. He also fed him the juice from tree leaves. After one and a half months, my son was completely cured. To cure my child, I had to visit the *kabiraj*, and he got better treatment compared to the hospital.

### Diagnostic symptoms and common treatments for *jaundeesh*

Since *kabirajs* were the most commonly-visited treatment providers, the interview team conducted in-depth interviews with 25 most commonly-visited *kabirajs* in the Matlab study area to understand symptoms used in diagnosing *jaundeesh* and the prescribed treatments. On average, the interviewed *kabirajs* were 54 years old, had been practising for 10 years, and provided treatment for *jaundeesh* to three patients per week. Of the 25 *kabirajs* interviewed, 48% recorded patients’ reports of foul smell in food and water as the main symptom that they used in diagnosing *jaundeesh/holde palong*. Loss of appetite was the main diagnostic symptom used by one-fifth of the *kabirajs* while another 20% cited dark-coloured urine. Only 12% mentioned yellow eyes or skin as the main diagnostic symptom of *jaundeesh.*

When asked about the types of treatments they prescribed for *jaundeesh*, more than half described ritual handwashing procedures known as *hat dhoa.* As described by a 44-year old *kabiraj* who had been practising for seven years, “*Hat dhoa* means actively washing out *jaundeesh*, using herbal medicines, such as tree bark.” During ritual handwashing, the *kabirajs* explained that yellow colouring was washed from the patients’ hands to remove the offending *jaundeesh* with water. A 60-year old *kabiraj* who had been practising for 14 years explained, “Patients said that they prefer *hat dhoa* since they directly see the yellow being washed away and the *jaundeesh* coming out in front of them.”

Table 3 shows the prescribed rituals and treatments for *jaundeesh.* Approximately one-fourth of the *kabirajs* recommended that patients wear sacred amulets around their neck, such as garlands or blessed items, to cure *holde palong*. One-fifth conducted ritual bathing of the body to remove *jaundeesh*. For children with *jaundeesh*, a 63-year old *kabiraj* who had been practising for 21 years explained:

Our wise elders say you must get up at dawn and dip the child in water every hour or so, or bathe the child in the pond 4-5 times a day so that he catches a cold. When he gets a cold (sneezing, runny nose), then you understand that the *jaundeesh* is running away.

*Kabirajs* commonly suggested that patients take herbal tablets or drink herbal mixtures. They described a variety of blessing rituals which they performed for their patients. Often, they performed a daily ritual at dawn at their home or their patient's home. For example, a 52-year old *kabiraj* who had been practising for 11 years explained his treatment process for children:

At dawn, I visit the sick child. After shaving the top of the head, I apply herbal paste at the top of the head every morning at dawn. I do this for three weeks to remove the *jaundeesh.*

**Table 3.** Typical treatments prescribed by *kabirajs* (n=25) for *jaundeesh/holde palong* in Matlab, Bangladesh, in 2003

Description of ritual	Percentage of *kabirajs* recommending this treatment*
Wash hands with herbal medicines or mixtures	56%
Wash out ‘yellow’ from patient's hands (‘*hat dhoa*’)	
Wash patient's hands with tree bark	
Wash hands with leaves from a vine (‘*lottapatha*’)	
Wash hands with water that has been standing overnight	
Wear amulet	24%
Twist garland (‘*mala’*) on head/neck	
Tie a banana with string, burn the banana, and tie the string to the patient's neck	
Perform bathing ritual	20%
Bathe with cold water until one gets a head cold to chase away jaundice	
Dunk patient in water repeatedly until nose begins to run	
Drink or apply herbal mixture	16%
Drink water in which a live mussel has been soaked overnight	
Shave head with a seashell and apply herbal paste	
Drink unpasteurized milk	
Consume symbolic item to remove jaundice	16%
Drink blessed water (*panipora*)	
Eat blessed bananas (*kolapora*)	
Eat blessed green coconut (*dabpora*)	
Eat blessed sour yoghurt (*dodhipora*)	
Place blessed paper-cone in ear and burn the cone	

*The total is greater than 100% since more than one treatment could be listed

## DISCUSSION

In the setting as ours in rural Bangladesh, one-fourth of surveyed respondents perceived having a condition they called *jaundeesh.* Two-thirds of these respondents lacked the visible sign of yellow eyes or skin, suggestive of clinical icterus. Despite reasonably-high sensitivity and specificity, the term *jaundeesh*, in this rural Bangladesh context, provided limited positive predictive value due to the high burden of self-reported *jaundeesh* among those lacking yellow eyes or skin. Respondents perceived *jaundeesh* to arise from humoral imbalances and largely sought for non-biomedical treatments from *kabiraj*, particularly methods which claimed to ‘wash out’ the *jaundeesh* from their skin. As the main treatment providers in this population, only 12% of *kabirajs* used the symptom of yellow eyes or skin to diagnose *jaundeesh.*

Nearly two-thirds of the respondents with *jaundeesh* reported visiting *kabirajs* for care. Previous studies in rural Bangladesh indicate that patients may seek local treatment providers, such as *kabirajs* due to their accessibility, lower cost, and flexibility in payment schemes (10). One study conducted on informal treatment providers in Matlab, Bangladesh, in 1978 suggested that patients reporting jaundice almost exclusively visited *kabirajs* and other traditional healers for care, reflecting that these care-seeking pathways may, perhaps, be deeply ingrained in rural populations (11). Most *kabirajs* are deeply embedded in rural communities and are called to visit homes for treatment, reflecting a high degree of community trust. *Kabirajs* generally prescribe the use of herbal remedies which have been used since at least 175 BC for treatment of jaundice in the Indian Subcontinent (19). Practices of faith healing rooted in ancient Animist religions and Pagan folk culture, and in rural areas, have been integrated into modern religious paradigms (15). Items believed to ward off evil spirits, such as amulets, local tree leaves, cow bones, iron knives, bamboo broomsticks, are commonly prescribed by *kabirajs* (20). While care-seeking is recognized as a complex behaviour influenced by many factors (21) in this rural population, *kabirajs* may offer a specialized niche in the rural healthcare market for provision of traditional treatments for perceived *jaundeesh* (8).

Most *kabirajs* inform patients that their symptoms will subside with their treatment after two to three weeks (22), a timespan which is co-incident with the self-limited natural history of most acute viral hepatitis. Patients report feeling that treatments, such as using amulets or washing away *jaundeesh* with spiritual water, have instant effects. While some treatments prescribed by *kabirajs* overlap with clinical recommendations, such as drinking plenty of fluids, most treatments prescribed for jaundice by *kabirajs* were non-biomedical. Some treatments were even potentially harmful, for example, one *kabiraj* recommended that his patient “wash his hands with water that has been standing for three days.” Then, the *kabiraj* advised him to “to drink unpasteurized milk with herbal medicine.” *Kabirajs* commonly advised patients to avoid meat and eggs, although protein intake is clinically recommended for optimal recovery (23). Practices such as these may hinder recovery or increase the risk of super-infection in patients already immunologically challenged by one infection.

Population-based studies that aim to use reported morbidities to detect hepatitis-like illnesses may benefit from an understanding that *jaundeesh* in the absence of visible signs of icterus, reported by two-thirds of our respondents, may be commonly perceived in this population. Given the degree of community trust and extant explanatory mechanisms for this illness, *kabirajs* are likely to remain first-line care providers for perceived *jaundeesh* in rural communities. Inclusion of *kabirajs* for health education and training may be useful to improve patient outcomes through referral of patients for whom immediate referral to clinics is warranted, such as cases of possible hepatitis E infections in pregnancy, where the prognosis is exceptionally poor and where clinical supportive management may reduce the risk of mortality (4).

This study was carried out on a random selection of rural residents and *kabirajs* in Matlab, an area of Bangladesh, generally representative of rural Bangladesh. Findings may be culturally unique to this study population, although anecdotal reports from Nepal suggest that similar local definitions are used for the word jaundice (Labrique, personal communication, 2012). Although additional interviews with respondents reporting *jaundeesh* as well as clinical signs of icterus might have allowed for comparison, this study focused on perceptions of *jaundeesh* among those lacking icterus in order to highlight local illness perceptions that may inform future population-based studies on reported illness symptoms.

### Conclusions

*Jaundeesh/holde palong* is commonly recognized in this rural Bangladesh population as a distinct syndrome among anicteric respondents. *Jaundeesh* is associated with a number of signs and symptoms, some of which differ from the biomedical definition of jaundice. In this setting, *kabirajs* are the main care providers for *jaundeesh* and offer multiple treatment options, ranging from benign rituals to potentially harmful practices. Further research on the aetiology of hepatitis-like illness in this population should account for the local perceptions of the disease entity known as *jaundeesh*. An understanding of the cultural perceptions of *jaundeesh* may inform future population-based epidemiological surveys for surveillance of reported symptoms in populations of rural Bangladesh. These data may also inform national programmes for hepatitis prevention and treatment strategies, such as introduction of hepatitis B vaccination, and improve public-health strategies for surveillance, detection, and response.

## ACKNOWLEDGEMENTS

This study was funded by NIH-NIAID grant (1 R01 AI51/31/2004). icddr,b acknowledges with gratitude the commitment of NIH-NIAD, USA to its research efforts. The authors would like to acknowledge the dedication and hard work of the HEV-Matlab field team (Bashiruddin Ahmad, S.R. Paul, A.B.M. Borhan Uddin, Nasrin Akter, Nurunnahar Lina, and Sabina Akhter) as well as the administration and staff of icddr,b for their support.
